# Echinocystic acid inhibits the growth of human bladder cancer cells by suppressing AKT1/GSKβ/β-Catenin signaling and synergizes with gemcitabine

**DOI:** 10.1016/j.gendis.2026.102148

**Published:** 2026-03-19

**Authors:** Xueqian Han, Liping Xie, Ziyun Li, Yajun Hou, Yunfeng He, Liqiong He, Jinyong Luo

**Affiliations:** a College of Laboratory Medicine, Chongqing Medical University, Key Laboratory of Clinical Laboratory Diagnostics (Chinese Ministry of Education), Chongqing 400016, China; b Department of Urology, The First Affiliated Hospital of Chongqing Medical University, Chongqing 400016, China

**Keywords:** AKT1, Bladder cancer, Echinocystic acid, Ferroptosis, Natural product

## Abstract

Bladder cancer (BC) is an aggressive and treatment-resistant malignancy with a high recurrence rate. Current first-line therapies only provide limited relief due to chemoresistance and toxicity. Natural products are emerging as promising chemotherapeutic agents due to their multi-target mechanisms and excellent safety profile. In this study, we evaluated the anti-BC effects of echinocystic acid (EA), a pentacyclic triterpenoid, on BC cells and investigated the underlying mechanism. We found that EA effectively inhibited proliferation, triggered G1-phase cell cycle arrest, and simultaneously suppressed cellular invasion and migration capabilities *in vitro*. Although EA did not obviously induce apoptosis in BC cells at the doses that were effective in suppressing their growth, it successfully triggered ferroptosis within these cells. Moreover, EA suppressed xenograft tumor growth of BC cells *in vivo* and demonstrated a favorable safety profile. Mechanistically, EA interacted with AKT1, reducing its phosphorylation and thereby inhibiting the AKT1/GSK3β/β-Catenin signaling pathway. Furthermore, EA exhibited synergistic effects with gemcitabine in inhibiting BC cells *in vitro* and *in vivo*. Overall, our results suggest that EA may exert its anti-BC effects, at least in part, by inhibiting the AKT1/GSK3β/β-Catenin signaling pathway. When combined with the clinically approved anti-tumor drug gemcitabine, EA may produce a synergistic anti-BC effect. Most importantly, this study identifies EA as a novel inducer of ferroptosis in BC and underscores its potential for clinical application.

## Introduction

Bladder cancer (BC) is the most prevalent malignant neoplasm of the urinary system, ranking ninth among all malignancies in global incidence rates.^1^ The core treatment modality for BC is currently surgical resection, with subsequent chemotherapy being an essential adjunct following surgery.^2^ Although adjuvant chemotherapeutic agents have improved outcomes, their inherent off-target toxicities, agonizing dose-limiting side effects, and acquired multiple drug resistance frequently undermine therapeutic efficacy, ultimately compromising patient survival. These enduring clinical challenges emphasize the imperative to develop novel therapeutic agents that operate through innovative mechanisms of action, offering enhanced efficacy while minimizing the risk of systemic toxicity.

Natural products are renowned for their multi-targeting capabilities and favorable safety profiles, making them a promising source for the discovery of next-generation anti-cancer agents. Echinocystic acid (EA) is a pentacyclic triterpenoid compound abundant in *Pueraria lobata*, exhibiting diverse pharmacological properties, including anti-HCV,^3^ anti-inflammatory,^4^ and anti-obesity effects.^5^ Notably, emerging evidence has also emphasized its exciting anti-tumor activity in multiple forms of malignancy, including ovarian cancer,^6^ leukemia,^7^ non-small cell lung cancer,^8^ and hepatocellular carcinoma.^9^ However, many issues concerning the anti-tumor effects of EA remain unaddressed. Firstly, the existing evidence is primarily from *in vitro* studies; thus, more *in vivo* research is required to confirm its effectiveness in combating tumors. Secondly, the anti-tumor efficacy of EA in other tumor types is unclear, making it difficult to confirm whether EA possesses broad-spectrum anti-tumor activity. Thirdly, the underlying molecular mechanisms of EA's anti-tumor effects remain incompletely understood.

Herein, we aimed to systematically evaluate the anti-tumor activity and delineate the mechanism of action of EA on human BC cells, and preliminarily explore the feasibility of achieving synergistic anti-BC effects through the combination of EA and gemcitabine (GEM). The results indicated that EA selectively suppressed the growth of BC cells *in vitro* and *in vivo*, while showing minimal cytotoxic effects on normal cells and demonstrating a favorable safety profile in immunocompetent mice. Mechanistic investigation revealed that EA triggered ferroptosis in BC cells by suppressing the AKT1/β-Catenin signaling axis. Furthermore, we observed a marked synergistic effect between EA and GEM in inhibiting BC cell growth.

## Materials and methods

### Cell culture and reagents

The following human cell types were employed in this study: T24 and EJ bladder carcinoma cell lines, SV-HUC-1 bladder epithelial cells, and HK-2 renal tubular cells. All cells were sustained in an appropriate medium enriched with 10% fetal bovine serum and incubated in a humidified incubator containing 5% CO_2_ at 37 °C. The main reagents were EA (98% purity; Chengdu Refines Biotechnology, China), GEM (MedChemExpress, USA), SC79 (Selleckchem, USA), and Ferrostatin-1 (Fer-1, Selleckchem, USA).

### Crystal violet staining

T24 and EJ cells were plated in 24-well plates at a density of 2.5 × 10^4^/well. After reaching approximately 30% confluency, these cells underwent treatment with EA at varying doses (0, 10, 20, 30, and 40 μM) for a duration of 24, 48, or 72 h. Post-treatment, the cells were first fixed with 4% paraformaldehyde for 30 min and subsequently stained with 0.5% crystal violet for another 10 min. After air-drying, the plates were scanned to obtain digital images. To quantify the results, the deposited dye was dissolved in 10% glacial acetic acid, and absorbance was recorded at 595 nm.

### CCK-8 viability assay

First, the four cell types (T24, EJ, SV-HUC-1, HK-2) were plated in 96-well plates at an initial cell density of 3 × 10^3^/well. After complete adhesion, these cells underwent treatment with EA at varying doses (0, 10, 20, 30, and 40 μM) for a duration of 24, 48, or 72 h. Next, 10 μL of CCK-8 working solution (TargetMol, China) was introduced into each well for a 2-h reaction period. The absorbance values were then read at a wavelength of 450 nm.

### Colony formation assay

T24 and EJ cells were plated in 6-well plates with an initial density of 1 × 10^3^/well. Once microcolonies consisting of approximately 3–4 cells had formed, the cells were exposed to EA for 7 days. Following fixation, the resulting colonies were stained with crystal violet. Quantitative colony counting was conducted using ImageJ software.

### Flow cytometry analysis

After reaching approximately 30% confluency in 6-well plates, the four cell types (T24, EJ, SV-HUC-1, HK-2) were subjected to a 24-h treatment with various doses of EA (0, 10, 20, 30, and 40 μM). Cells were then collected for flow cytometric evaluation of apoptosis and cell cycle progression. To identify apoptosis, cells were co-stained with Annexin V-FITC/propidium iodide after phosphate-buffered saline washing. To analyze the cell cycle, cells were fixed in pre-chilled 75% ethanol (4 °C overnight), followed by propidium iodide staining.

### Wound-healing assay

T24 and EJ cells were seeded in 6-well plates and cultured until a fully confluent cell monolayer formed. Using a sterile 10 μL pipette tip, two straight lines perpendicular to each other were scratched onto the cell monolayer in the north-south and east-west directions to form a cross shape. After scratching, cells were incubated in a medium without serum, containing various concentrations of EA according to the experimental requirements. The wound-healing capacity was quantitatively assessed using the following formula: wound-closure rate = (end-time point scratched area –0-h scratched area)/0-h scratched area × 100%.

### Transwell assay for invasion/migration assessment

In the invasion assay, the Matrigel underwent a 20-fold dilution first. Afterward, 60 μL of this diluted Matrigel was placed in the upper chamber and left to solidify at 37 °C for 30 min. Post-trypsinization, 2.5 × 10^4^ BC cells in serum-free medium were seeded into the upper chamber, whereas the lower chamber contained 500 μL of complete medium supplemented with EA. Following a 24-h treatment period, cells retained on the sub-membrane surface were fixed, stained with crystal violet, and imaged to enable quantitative counting. In parallel, the migration assay followed the same procedure, but excluded the Matrigel coating step.

### Western blotting

First, T24 and EJ cells were lysed using a lysis buffer that included 1% protease and phosphatase inhibitors. Afterward, the resulting lysate's total protein concentration was measured with a BCA kit (Beyotime, China), and this concentration was used to adjust the loading volume to ensure equal protein amounts across the samples. After mixing with loading buffer and for denaturation, proteins were separated by 10% SDS-PAGE electrophoresis, and then transferred onto methanol-preactivated PVDF membranes. After blocking the membranes with 5% non-fat milk, they were incubated overnight at 4 °C with primary antibodies that specifically bind to the target protein, followed by a 1-h incubation with secondary antibodies (ZSGB-BIO, China) at 37 °C. Subsequently, a chemiluminescent assay kit was employed to visualize the protein bands, and semi-quantitative analysis was performed with the ChemiDoc MP imaging system (BioRad, USA). The antibodies used are listed in Table S1.

### Network pharmacology

PharmMapper^10^ and SwissTargetPrediction^11^ were used to predict the potential targets for EA action. These targets were intersected with BC-associated targets from GeneCards^12^ and the Therapeutic Target Database (TTD)^13^ to generate common targets that appeared in all sources. These targets were then visualized using an online Venn diagram tool and were subsequently imported into the DAVID database^14^ for Kyoto Encyclopedia of Genes and Genomes (KEGG) pathway and Gene Ontology (GO) enrichment analyses.^15^ Additionally, a protein–protein interaction network was created using the STRING database^16^ and visualized with Cytoscape software (version 3.9.1).

### Molecular docking

The 3D configuration of the AKT1 protein and the 2D structure of EA were downloaded from the Protein Data Bank,^17^ and PubChem,^18^ respectively. The EA molecule and AKT1 protein were pre-processed using AutoDockTools (version 1.5.6) and PyMOL (version 2.2.0), which included removing water molecules, ligands, and ions, as well as incorporating hydrogen atoms. Molecular docking procedures were subsequently executed with PyRX software, and the resulting docked complexes were visualized with Discovery Studio.

### Molecular dynamics

Molecular dynamics simulations were performed using GROMACS 2022. Force field parameters were assigned using pdb2gmx for AKT1 (AMBER14SB force field) and AutoFF for EA (GAFF2 force field). The system was solvated in a 1 nm TIP3P cubic water box, with ions added for electrostatic neutrality.^19^ PME was used for long-range electrostatics with a 1 nm cutoff. The SHAKE algorithm constrained bonds, and the timestep was set to 1 fs using the Verlet leapfrog method. Energy minimization occurred in steps: first, minimizing the solvent with a constrained solute, then ions with constraints, and finally, optimizing the full system. A 100 ns NPT simulation was performed at 310 K, analyzing parameters such as root mean square deviation (RMSD), root mean square fluctuation (RMSF), and radius of gyration (Rg).

### RNA sequencing

T24 cells were seeded at 30% confluence in 6 cm culture dishes and treated with either 20 μM of EA or DMSO for 24 h. Following media removal, 1 mL of Trizol reagent was added, mixed for 5 min, and pipetted repeatedly to ensure complete cell lysis. Then, the lysates were transferred to cryovials and submitted to LC-Bio Technology (China) for RNA sequencing, with each group comprising three biological replicates. Differentially expressed genes were identified and functionally characterized using KEGG pathway analysis and Gene Set Enrichment Analysis (GSEA).

### Reactive oxygen species (ROS) detection

Following 24-h pre-treatment with EA, T24 cells were mixed with 10 μM DCFH-DA (Beyotime, China), a ROS-specific fluorescent dye, for 30 min. Subsequent to rinsing with phosphate buffered solution (PBS), the observation of ROS-dependent green fluorescence was performed immediately using a fluorescence microscope.

### Lipid peroxidation assessment

Upon completion of the scheduled treatment, the cells were incubated with the BODIPY 581/591 C11 probe (Beiren Chem, China) at 37 °C for 30 min. The shift in fluorescence from red to green was captured using a confocal microscope. For co-localization analysis of mitochondria and lipid peroxidation, the cells were sequentially stained with MitoTracker Green FM working solutions (200 nM; Yeasen, China) at 37 °C for 30 min and then with MitoSOX Red Mitochondrial Superoxide Indicator working solutions (500 nM; Yeasen, China) for 20 min, with PBS washes between incubations. Images were acquired using confocal microscopy to assess spatial correlation.

### Glutathione (GSH)/oxidized glutathione (GSSG) ratio determination

Briefly, T24 cells were lysed and deproteinized, and the total glutathione (GSH plus GSSG) in the supernatant was first quantified. Next, after the elimination of GSH with the clearing reagent, the remaining GSSG was measured quantitatively. The GSH level was determined according to the formula: GSH = total glutathione – 2 × GSSG. GSH/GSSG ratio calculation was performed subsequently.

### Quantification of malondialdehyde (MDA) content

After 24 h of EA pretreatment, T24 cells were subjected to lysis and centrifugation. The resulting supernatant was then incubated at 100 °C with thiobarbituric acid for 15 min, and the absorbance value was read at a wavelength of 532 nm. MDA levels were normalized to the total protein content.

### Transmission electron microscopy

T24 cells were stimulated with EA either alone or in combination with Fer-1 (1 μM), SC79 (10 μM), or β-Catenin-expressing adenovirus (Ad-β-Catenin). Twenty-four hours after stimulation, the cells were trypsinized and then centrifuged to harvest cell masses. After fixation, the cells were stained and then subjected to transmission electron microscopy analysis.

### BC cell-derived xenograft tumor model

The ARRIVE guidelines were followed for all animal procedures, and approval and supervision were granted by the CQMU Ethics Committee (IACUC-2024-0003). Female BALB/c nude mice, aged 4 weeks, received subcutaneous injections of T24 cells (1 × 10^7^/injection) to generate a xenograft tumor model. Treatments, including sodium carboxymethyl cellulose (CMC-Na) or various concentrations of EA, were delivered orally by gavage every two days. Throughout the experimental period, tumor size and animal body weight were recorded. After finishing 10 rounds of treatment, the animals were euthanized through cervical dislocation. Subsequently, xenograft tumors and key organs (heart, liver, spleen, lungs, kidneys) were harvested for pathological assessment, such as hematoxylin–eosin staining and immunohistochemical examination.

### *In vivo* safety assessment

Four-week-old female immunocompetent BALB/c mice were acclimated for one week under specific pathogen-free conditions before being randomly assigned into five experimental groups: a CMC vehicle control group and four EA treatment groups receiving doses of 0, 20, 40, and 60 mg/kg. Treatments were delivered by oral gavage on alternate days. Body weight was recorded before and after each treatment. Terminal blood was collected through retro-orbital puncture for complete blood counts, liver/kidney function profiling, and blood smear analysis. After euthanasia, major organs were harvested for hematoxylin–eosin staining.

### Hematoxylin–eosin staining and immunohistochemical examination

Paraffin-embedded sections of 5 μm thickness were deparaffinized in xylene and rehydrated using graded ethanol. Hematoxylin–eosin staining and immunohistochemical examination were conducted following standard procedures. Representative images were observed and captured using an optical microscope.

### Drug combination assessment

Drug interactions were depicted using SynergyFinder 2.0 (https://synergyfinder.org/), considering a ZIP synergy score > 10 as indicative of a strong interaction. Complementary analysis was conducted using Jin's formula: *Q* = E_(a+b)_/(E_a_ + E_b_ – E_a_ × E_b_), where *Q* values below 0.85 signified antagonistic effects, values between 0.85 and 1.15 represented additive effects, and values equal to or greater than 1.15 indicated synergistic interactions.

### Statistical analysis

The data were expressed as mean ± standard deviation from a minimum of three independent experiments. Differences among three or more groups were determined using a one-way ANOVA, while differences between two groups were evaluated using Tukey's test. A *P*-value below 0.05 was defined as statistically significant.

## Results

### EA suppresses BC cell proliferation and induces G1 phase arrest

To evaluate the anti-tumor potential of EA (chemical formula C_30_H_48_O_4_, PubChem CID 73309; see Fig. 1A for the structure) against BC cells, its influence on cell proliferation was initially investigated. Crystal violet staining showed that EA caused a dose-dependent decrease in the number of attached cells (IC50 = 18.7 μM; Fig. 1B). Meanwhile, CCK-8 assays revealed that cell survival decreased noticeably following exposure to EA (Fig. 1C). Moreover, colony formation assays indicated that EA strongly suppressed the clonogenic potential of BC cells (Fig. 1D). At the protein level, EA was observed to reduce the expression of the proliferation markers PCNA (a regulator of DNA replication) and c-Myc (Fig. 1E), providing further molecular evidence of its antiproliferative activity. Furthermore, EA treatment also caused a substantial increase in the proportion of cells arrested in the G1 phase (Fig. 1F). In line with this, Cyclin D1, a critical driver of the G1/S transition,^20^ was reduced accordingly (Fig. 1G). These results together support a potential role for EA in inhibiting proliferation and triggering G1 phase arrest in BC cells. However, it is noteworthy that, despite EA having been demonstrated to induce apoptosis in several types of cancer cells,^7^, ^8^, ^9^ we did not observe any noticeable apoptosis-inducing effects of EA on BC cells following 24 h of treatment within the tested dose range (10–40 μM) (Fig. S1).

**Figure 1:**
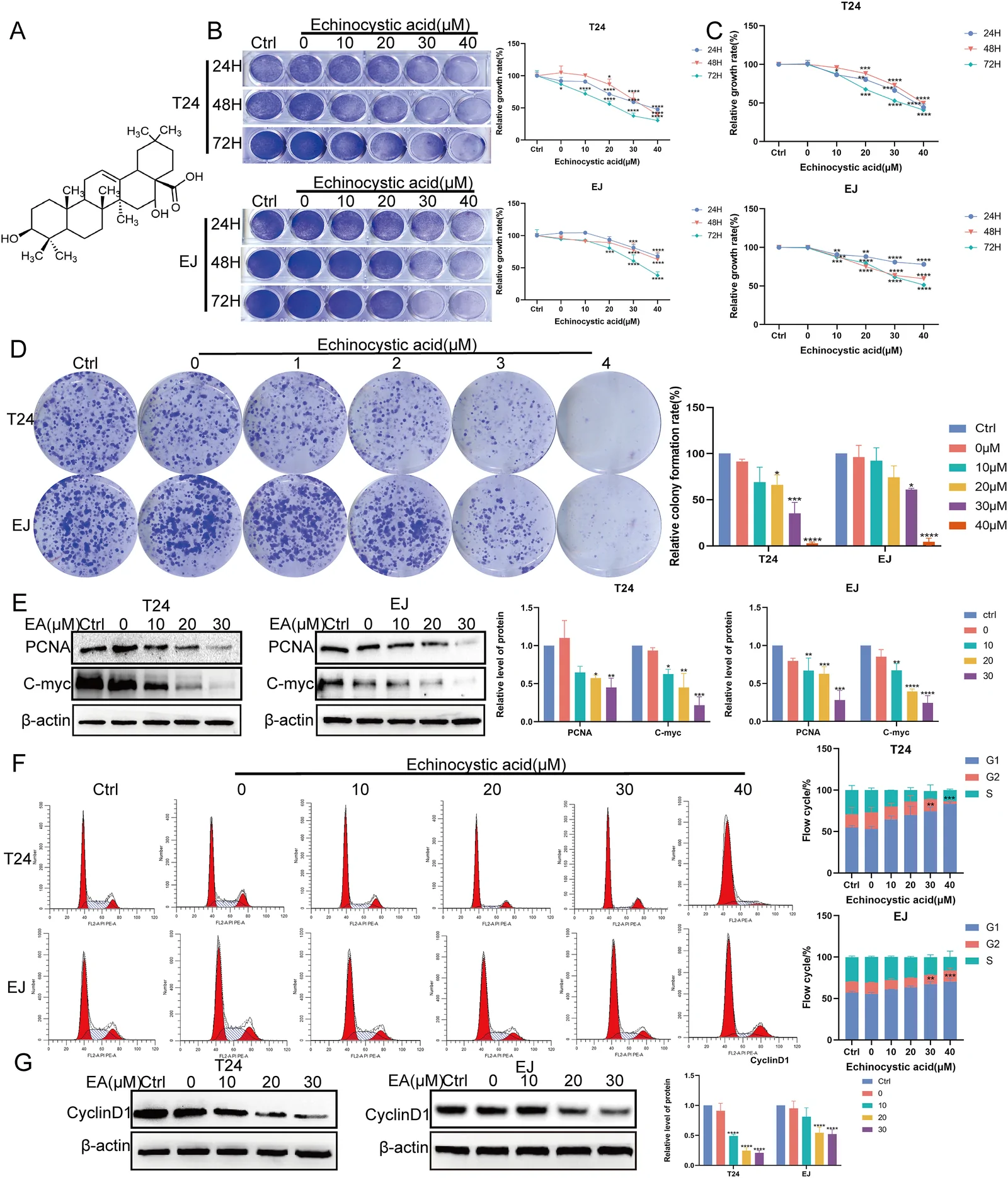
Echinocystic acid (EA) inhibits proliferation and induces G1 cell cycle arrest of breast cancer cells. **(A)** Two-dimensional chemical structure of EA. **(B)** Crystal violet staining indicated that EA assessed the cell adhesion survival rate of breast cancer cells (*n* = 3). **(C)** CCK-8 assay was performed to confirm the anti-proliferative effect of EA (*n* = 3). **(D)** Cloning assay showed that EA significantly inhibited the colony formation ability of cells (*n* = 3). **(E)** Western blotting assay showed that EA treatment significantly down-regulated the expression of proliferation marker proteins (*n* = 3). **(F)** Flow cytometry analysis revealed that EA caused G1 phase arrest (*n* = 3). **(G)** Western blotting showed that the expression of G1 phase regulatory protein cyclinD1 was significantly down-regulated (*n* = 3). The data are displayed as mean ± standard deviation. ∗*p* < 0.05, ∗∗*p* < 0.01, ∗∗∗*p <* 0.001, and ∗∗∗∗*p* < 0.0001.

### EA impairs BC cell migration and invasion

We next evaluated the influence of EA on the metastatic behavior of BC cells. EA-treated BC cells exhibited delayed wound closure in wound-healing assays (Fig. 2A) and a dose-dependent decrease in migrating cell members in Transwell migration assays (Fig. 2B). This suggests that EA hinders cell migration. Furthermore, EA markedly suppressed the invasiveness of BC cells, demonstrating a significant decrease in the number of cells penetrating Matrigel-coated membranes in invasion assays (Fig. 2C). Importantly, Western blotting analysis revealed that EA reversed epithelial–mesenchymal transition, a critical process in metastasis,^21^ causing an increase in E-cadherin levels and a decrease in N-cadherin and Vimentin levels (Fig. 2D). Likewise, the protein level of MMP-2, a critical matrix metalloproteinase (MMP) executing extracellular matrix degradation and facilitating cancer invasion,^22^ was also diminished following EA administration. Therefore, it appears that EA may disrupt the metastatic potential of BC cells.

**Figure 2:**
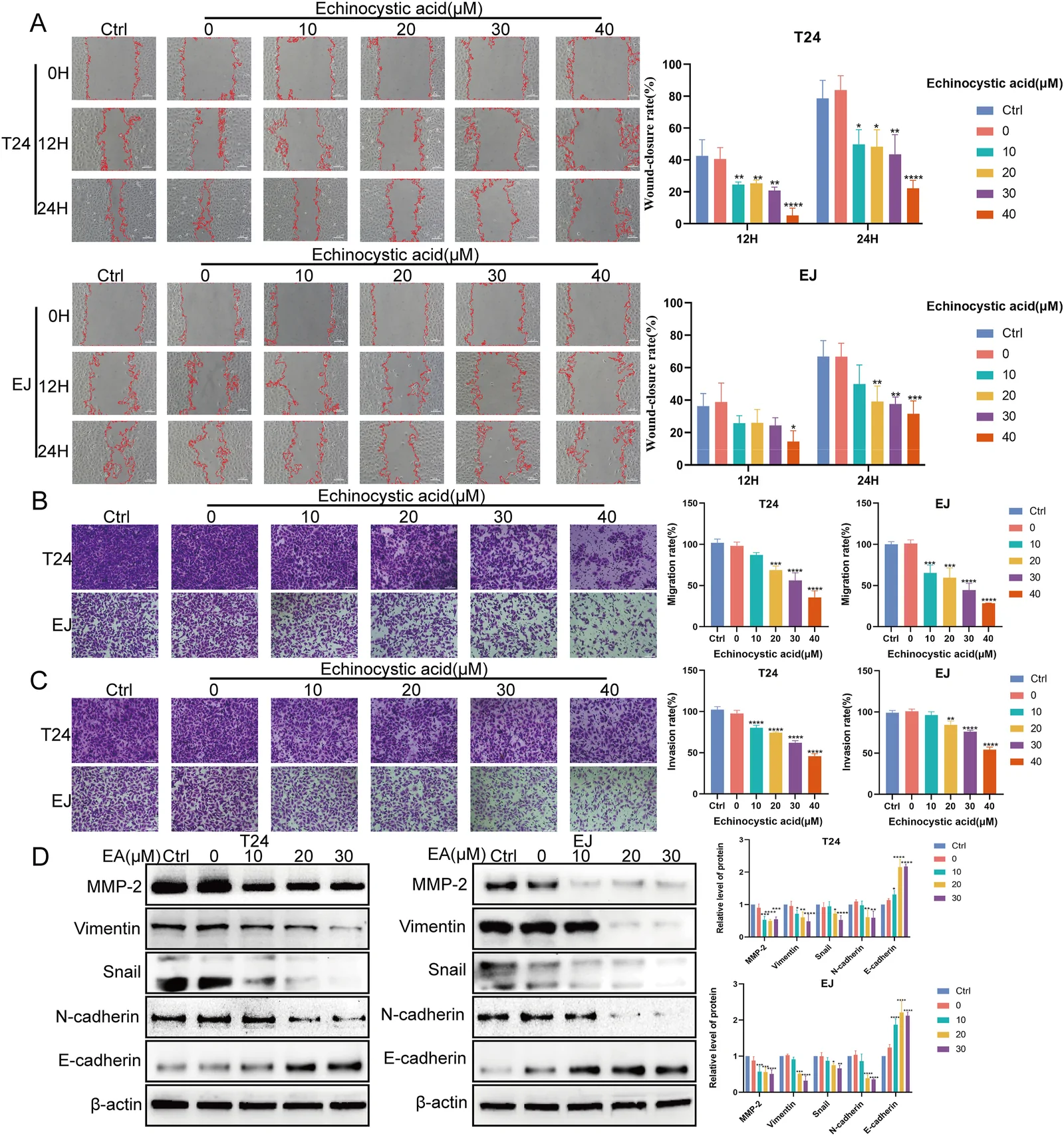
Echinocystic acid (EA) inhibits the migration and invasion of human breast cancer cells. **(A)** Scratch assay showed that EA treatment significantly delayed wound healing (*n* = 3). **(B)** Transwell migration assay quantitatively showed that the number of transmembrane cells in the EA treatment group was significantly reduced (*n* = 3). **(C)** Matrigel invasion assay showed that EA significantly inhibited cell invasion ability (*n* = 3). **(D)** Western blotting analysis showed that EA treatment significantly up-regulated the epithelial marker E-cadherin, while down-regulating the mesenchymal markers N-cadherin and MMP-2 (*n* = 3). The data are displayed as mean ± standard deviation. ∗*p* < 0.05, ∗∗*p* < 0.01, ∗∗∗*p <* 0.001, and ∗∗∗∗*p* < 0.0001.

### EA suppresses xenograft tumor growth of BC cells *in vivo*

In xenograft tumor models, the administration of EA successfully inhibited the *in vivo* growth of tumors formed by BC cells (Fig. 3A and B), with no accompanying loss in body weight (Fig. 3C). Subsequent hematoxylin–eosin staining revealed increased nuclear condensation and reduced nuclear-to-cytoplasmic ratios in EA-treated xenograft tumors (Fig. 3D). Consistent with this observation, immunohistochemical analysis showed decreased expression of proliferation and metastasis-related markers, including PCNA, Vimentin, and MMP2 (Fig. 3E). These results corroborate that EA possesses the potential to prevent BC cells from forming tumors *in vivo*. Noticeably, histopathological assessment of liver and kidney tissues from EA-administered nude mice presented no evident drug-induced damage, even at higher concentrations (Fig. 3F), suggesting a favorable preliminary safety profile for EA.

**Figure 3:**
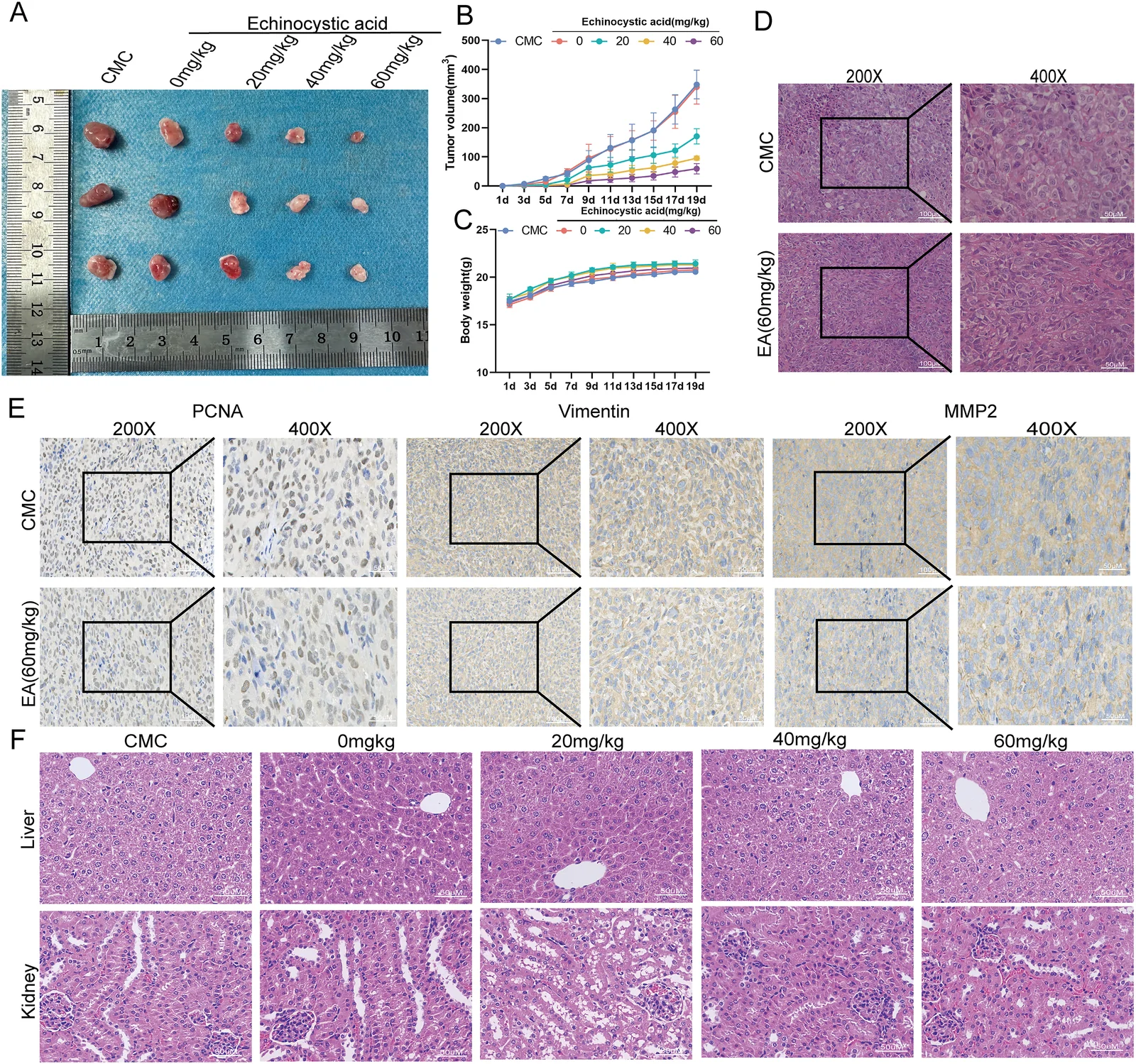
*In vivo* anti-tumor efficacy of echinocystic acid in breast cancer cell-derived xenograft tumor model. **(A)** Representative images of xenograft tumors. **(B)** Tumor growth curve (*n* = 3). **(C)** Body weight changes of nude mice during treatment (*n* = 3). **(D)** Representative images of hematoxylin–eosin staining of tumor tissues (magnification: 200 × and 400 ×). **(E)** Representative images of immunohistochemical analysis of tumor tissues (magnification: 200 × and 400 ×). **(F)** Representative images of hematoxylin–eosin staining of liver and kidney tissues (magnification: 200 × and 400 ×). The data are displayed as mean ± standard deviation. ∗*p* < 0.05, ∗∗*p* < 0.01, ∗∗∗*p <* 0.001, and ∗∗∗∗*p* < 0.0001.

### EA exhibits favorable biosafety profiles

We then further assessed the biosafety of EA. In normal cells (HK-2 and SV-HUC-1) derived from the urinary system, EA treatment did not show any detectable anti-proliferative activity and apoptosis-inducing ability (Fig. 4A–D). We next examined the safety profile of EA in immunocompetent mice following the experimental process illustrated in Figure 4E (created with BioGDP.com).^23^ Mice receiving EA treatment maintained stable body weight throughout the experimental period (Fig. 4F). Apart from a moderate rise in platelet count at higher EA doses, no significant alterations were observed in any of the other blood routine parameters analyzed (Fig. 4G–J). Additionally, serum biochemical markers reflecting hepatic and renal function were also unaltered (Fig. 4K and L). Furthermore, histological assessment of the principal organs, specifically the liver, kidneys, heart, spleen, and lungs, revealed well-preserved tissue architecture and absence of evident histopathological changes indicative of toxicity (Fig. 4M and N). Overall, these findings support the favorable biosafety profile of EA, although its platelet-elevating effect requires further investigation.

**Figure 4:**
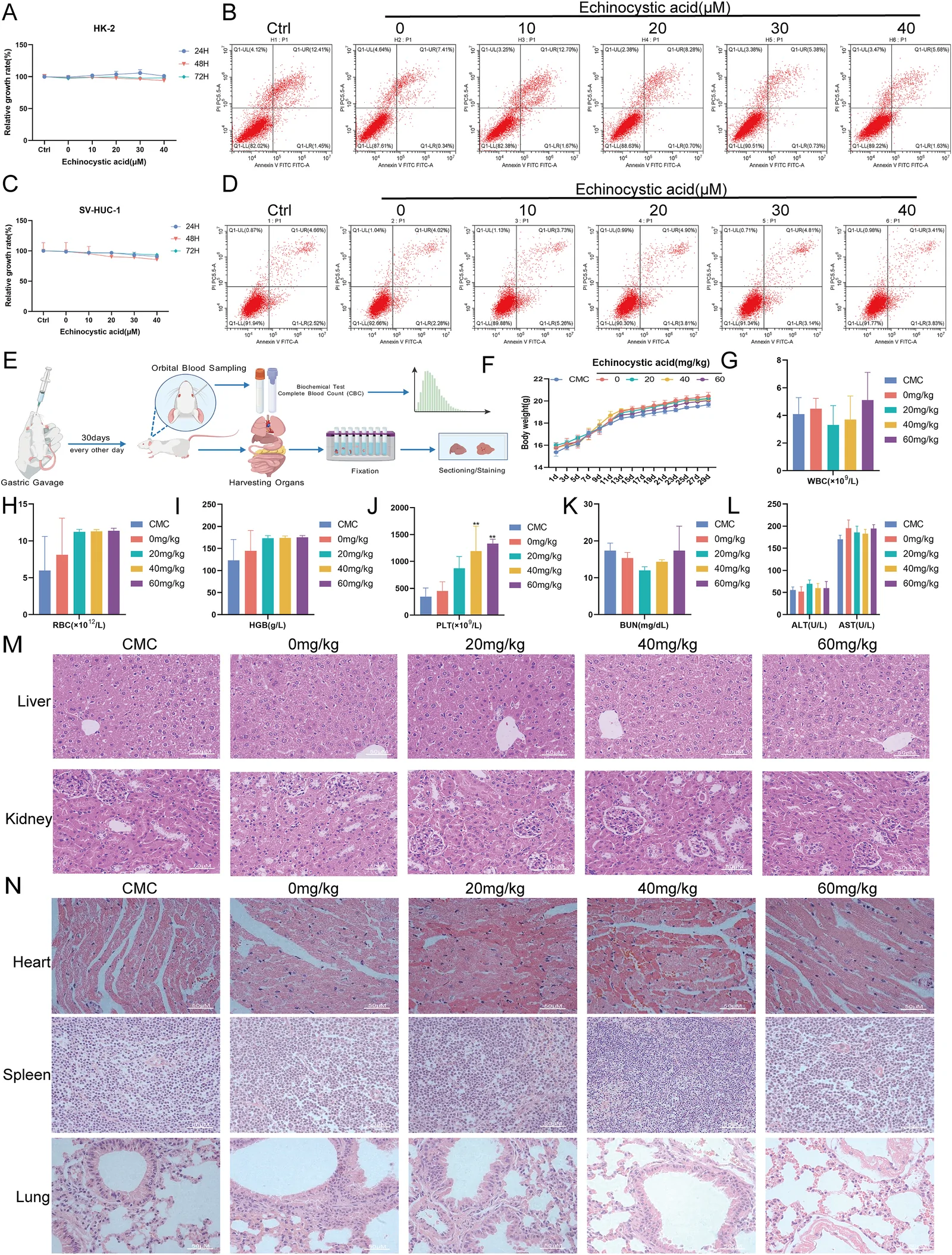
*In vitro* and *in vivo* biosafety assessment of echinocystic acid (EA). **(A, B)** Effects of EA (0–40 μM, 24 h) on HK-2 cells were determined by CCK-8 cell viability analysis (A) and flow cytometry apoptosis analysis (B) (*n* = 3). **(C, D)** Effects of EA (0–40 μM, 24 h) on HUC-1 cells were determined by CCK-8 cell viability analysis (C) and flow cytometry apoptosis analysis (D) (*n* = 3). **(E)** Schematic of *in vivo* safety evaluation protocol, created with BioGDP.com.^23^**(F)** Body weight trajectories of BALB/c mice during 29-day EA gavage (*n* = 3). **(G**–**J)** Complete blood count, including WBC (G), RBC (H), HGB (I), and PLT (J), was quantitatively analyzed after EA treatment (*n* = 3). **(K, L)** Serum biochemistry, including hepatic function (ALT, AST) (L) and renal function markers (BUN) (K), was quantitatively analyzed after EA treatment (*n* = 3). **(M, N)** Representative images of hematoxylin–eosin staining of major organs, including liver and kidney (M), heart, spleen, and lung (N) (magnification: 200 × and 400 ×). The data are displayed as mean ± standard deviation. ∗*p* < 0.05, ∗∗*p* < 0.01, ∗∗∗*p <* 0.001, and ∗∗∗∗*p* < 0.0001.

### EA inhibits AKT1/GSK3β/β-Catenin signaling in BC cells

Network pharmacology analysis yielded 251 overlapping targets between EA and BC (Fig. 5A). Within the protein–protein interaction network, AKT1 and ALB were identified as central hubs, with GSK3β also occupying a critical position (Fig. 5B). Given that the PI3K–AKT signaling pathway exhibited a consistent enrichment trend in both network pharmacology (Fig. 5C) and RNA sequencing analysis (Fig. 5D), AKT1 was prioritized for further validation. Molecular docking demonstrated a high-affinity binding (ΔG = −7.3 kcal/mol) between EA and AKT1 (PDB: 4EKL) (Fig. 5E and F). The predicted binding mode was structurally reliable, as supported by the Ramachandran plot showing 97.9% of residues in favored or allowed regions (Fig. 5G). Furthermore, molecular dynamics simulations indicated stable conformational behavior of the EA–AKT1 complex, evidenced by low and stable Rg, RMSD, and RMSF values (Fig. 5H–J). Cellular thermal shift assay further corroborated the direct interaction of EA and AKT1 in BC cells, showing a significantly enhanced thermal stability of AKT1 following EA treatment (Fig. 5K). Subsequently, Western blotting analysis demonstrated that EA treatment suppressed the phosphorylation of AKT1 (p-AKT1) at Ser473 and the phosphorylation of GSK3β (p-GSK3β) at Ser9, and reduced the abundance of β-Catenin protein in BC cells (Fig. 5L). The AKT1 agonist SC79 rescued the phosphorylation levels of GSK3β and β-Catenin from EA-induced suppression (Fig. 5M), demonstrating that the AKT1/GSK3β/β-Catenin signaling axis was operative. Consistently, a notable reduction in the phosphorylation levels of AKT1 and GSK3β, along with diminished β-Catenin expression, was observed in xenograft tumor tissues derived from BC cells following EA administration (Fig. 5N). Collectively, these findings imply that EA disrupts the AKT1/GSK3β/β-Catenin signaling axis in BC cells.

**Figure 5:**
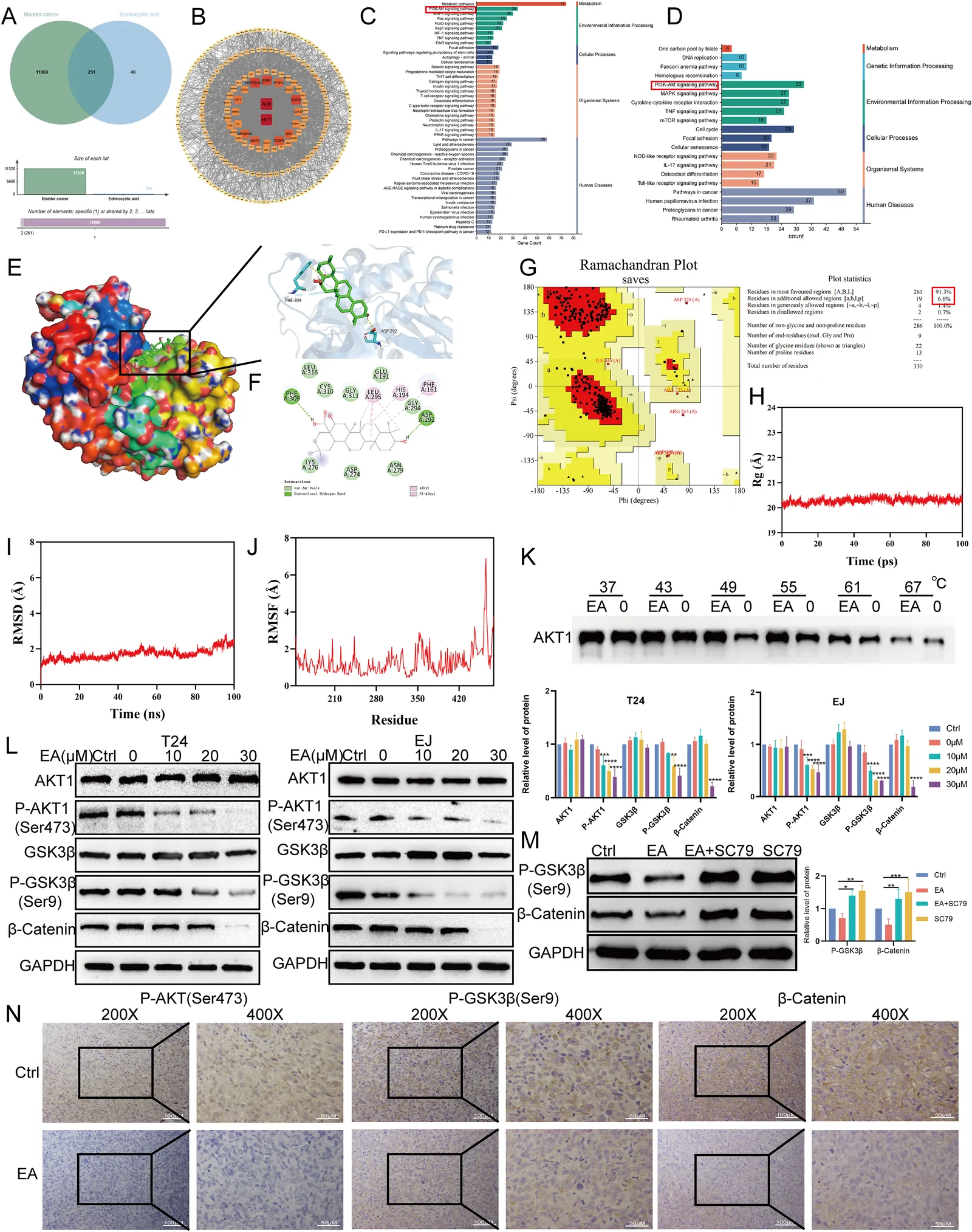
Network pharmacology and transcriptome sequencing reveal that echinocystic acid (EA) inhibits AKT1/GSK3β/β-Catenin signaling in breast cancer cells. **(A)** Venn diagram showing 251 overlapping targets between EA and breast cancer. **(B)** Protein–protein interaction (PPI) network of the overlapping targets. **(C)** KEGG enrichment analysis of network pharmacology. **(D)** KEGG enrichment analysis of RNA sequencing. **(E)** Molecular docking model of EA with AKT1. **(F)** The 2D structure of the molecular docking model. **(G)** The Ramachandran plot of the EA-AKT1 interaction was produced directly by the SAVES server. **(H**–**J)** The Molecular dynamics parameters of EA/AKT1 complexes, including Rg (H), RMSD (I), and RMSF (J), were analyzed using GROMACS 2022. **(K)** Cellular thermal shift assay demonstrated the temperature-dependent binding of EA to breast cancer cells. **(L)** Western blotting analysis of the essential molecules of the AKT1/GSK3β/β-Catenin pathway in EA-treated cells (*n* = 3). **(M)** Western blotting analysis of AKT1/GSK3β/β-Catenin pathway upon EA treatment in the presence of SC79 (*n* = 3). **(N)** Representative images of immunohistochemical analysis of tumors (magnification: 200 × and 400 × ). The data are displayed as mean ± standard deviation. ∗*p* < 0.05, ∗∗*p* < 0.01, ∗∗∗*p <* 0.001, and ∗∗∗∗*p* < 0.0001.

### EA induces ferroptosis in BC cells

GSEA of RNA sequencing data revealed significant enrichment of ferroptosis-related gene sets in EA-treated BC cells (Fig. 6A and B). Correspondingly, EA treatment down-regulated the levels of master ferroptosis controller, GPX4, and SLC7A11, in both BC cells (Fig. 6C) and xenograft tumors (Fig. 6D). To functionally verify the induction of ferroptosis, we introduced Fer-1, a specific inhibitor of this process. Pre-treatment with Fer-1 substantially restored the viability of BC cells following exposure to EA (Fig. 6E and F), suggesting a possible role of ferroptosis in EA-induced cell death. EA treatment initiated molecular alterations associated with ferroptosis, notably ROS accumulation (Fig. 6G), increased mitochondrial superoxide production (Fig. 6H), and enhanced intracellular lipid peroxidation (Fig. 6I). Ultrastructural observations by transmission electron microscopy unveiled that the mitochondria in some EA-treated BC cells exhibited increased membrane density, mitochondrial shrinkage, and loss of the cristae structure (Fig. 6J), which are precisely the hallmark changes occurring in mitochondria during ferroptosis.^24^ Biochemically, EA significantly reduced the GSH/GSSG ratio and increased MDA content. However, these characteristic ferroptosis events induced by EA were both counteracted by Fer-1 treatment (Fig. 6K and L). *In vivo*, Fer-1 partially attenuated the suppression of xenograft tumor growth by EA treatment (Fig. 6M). Subsequent hematoxylin–eosin staining showed a higher nuclear-to-cytoplasmic ratio in the Fer-1/EA co-treatment group compared with the EA-only group (Fig. 6N), implying that Fer-1 may restore the malignant cellular phenotype inhibited by EA. These results collectively suggest that EA is capable of initiating ferroptosis in BC cells.

**Figure 6:**
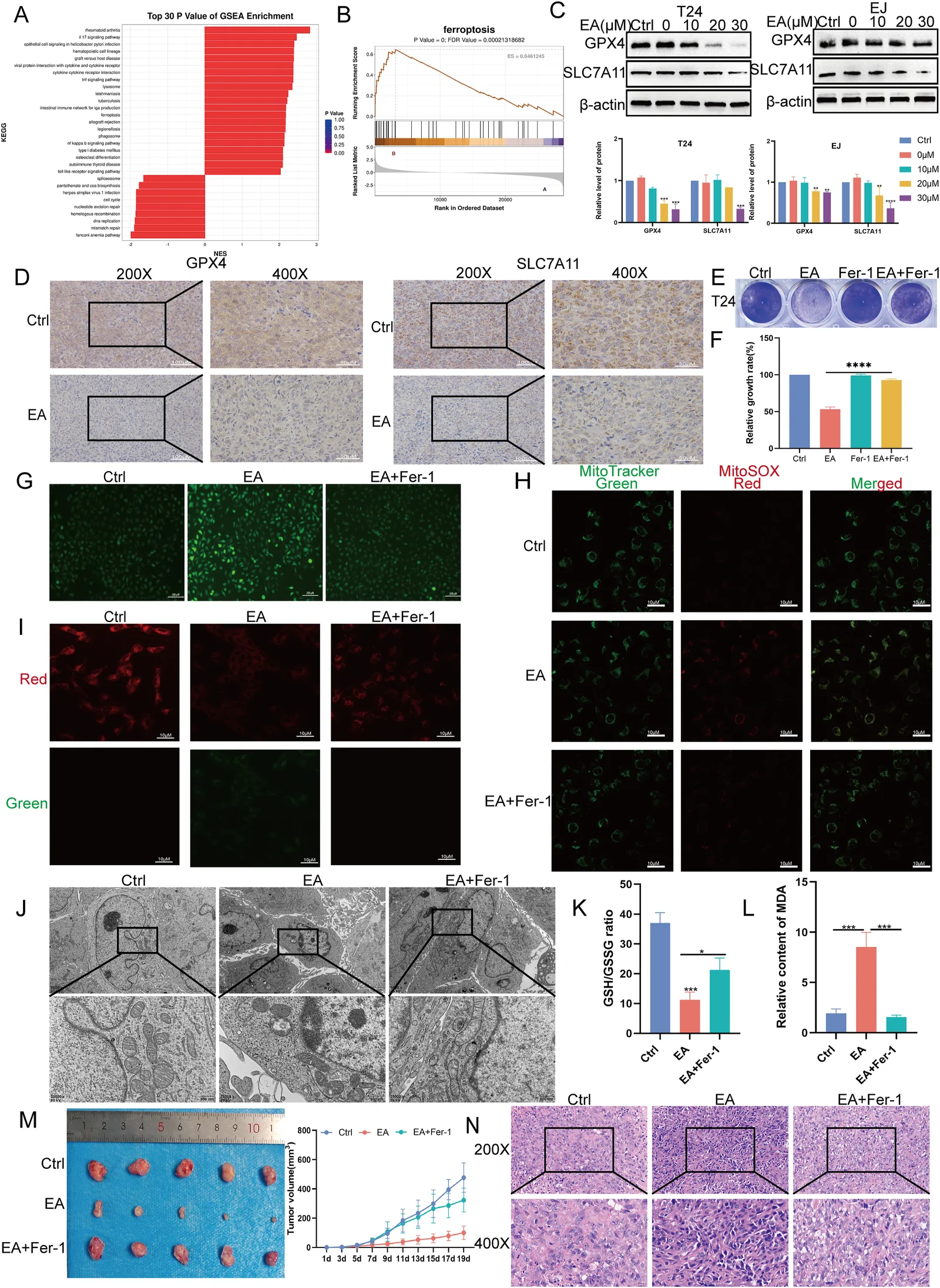
Echinocystic acid (EA) induces ferroptosis in breast cancer cells. **(A)** Gene Set Enrichment Analysis (GSEA) of transcriptome sequencing data post-EA treatment. **(B)** Ferroptosis pathway enrichment map. **(C)** Western blotting analysis of ferroptosis-related proteins of EA-treated breast cancer cells (*n* = 3). **(D)** Immunohistochemical analysis of ferroptosis-related proteins of xenograft tumor samples (magnification: 200 × and 400 ×). **(E)** Crystal violet staining showed that Fer-1 rescued EA-impaired T24 cell viability (*n* = 3). **(F)** CCK-8 assay showed that Fer-1 rescued EA-impaired T24 cell viability (*n* = 3). **(G)** DCFH-DA probe detected an increase in intracellular ROS levels in EA-treated T24 cells. **(H)** MitoTracker Green/MitoSOX Red staining revealed an increased mitochondrial lipid peroxidation in EA-treated T24 cells. **(I)** BDP-581/591-C11 probe detected an increased lipid peroxidation in EA-treated T24 cells. **(J)** Transmission electron microscopy of mitochondrial ultrastructure (magnification: 6000 × and 25000 ×). Ferroptosis is typically characterized by mitochondrial shrinkage, increased membrane density, and a reduction or disappearance of mitochondrial cristae. **(K)** EA treatment caused a GSH depletion and an increased GSSG/GSH ratio (*n* = 3). **(L)** EA treatment caused an elevated MDA level (*n* = 3). **(M)***In vivo* tumor image and growth curves (*n* = 5). **(N)** Representative images of hematoxylin–eosin staining of xenograft tumors (magnification: 200 × and 400 ×). The data are displayed as mean ± standard deviation. ∗*p* < 0.05, ∗∗*p* < 0.01, ∗∗∗*p <* 0.001, and ∗∗∗∗*p* < 0.0001.

### EA inhibits the proliferation, migration, and invasion of BC cells, while inducing ferroptosis, possibly via suppression of AKT1/GSK3β/β-Catenin signaling

In rescue experiments, administration of the AKT1 agonist SC79 effectively counteracted the suppressive effects of EA on cellular proliferation (Fig. 7B and C), migration, and invasion (Fig. 7D and E). As β-Catenin serves as the pivotal molecular hub linking the AKT1/GSK3β/β-Catenin pathway to the ferroptosis execution mechanism,^25^ we introduced an adenovirus overexpressing β-Catenin (Ad-β-Catenin; Fig. 7A) and discovered that this adenovirus produced effects analogous to those of the AKT1 activator SC79 (Fig. 7B–E). Moreover, SC79 and Ad-β-Catenin both impaired ferroptosis-related phenotypes induced by EA, including characteristic mitochondrial ultrastructure, the GSH/GSSG ratio, and MDA content (Fig. 7F–H). Concurrently, EA-induced alterations in protein expression of markers associated with proliferation (PCNA), invasion (Vimentin, MMP2), and ferroptosis (GPX4, SLC7A11) were reversed by SC79 and Ad-β-Catenin (Fig. 7I and J), confirming the involvement of the AKT1/GSK3β/β-Catenin pathway axis in EA monotherapy. *In vivo*, SC79 and Ad-β-Catenin markedly attenuated the anti-tumor efficacy of EA, resulting in a partial recovery of tumor volume (Fig. 7K) and a re-elevated nuclear-to-cytoplasmic ratio in hematoxylin–eosin-stained tumor sections (Fig. 7L). Finally, SC79 and Ad-β-Catenin restored the levels of GPX4 and SLC7A11, which had been suppressed by EA (Fig. 7M), thereby further underscoring the essential role of the AKT1/GSK3β/β-Catenin pathway in mediating EA-induced ferroptosis.

**Figure 7:**
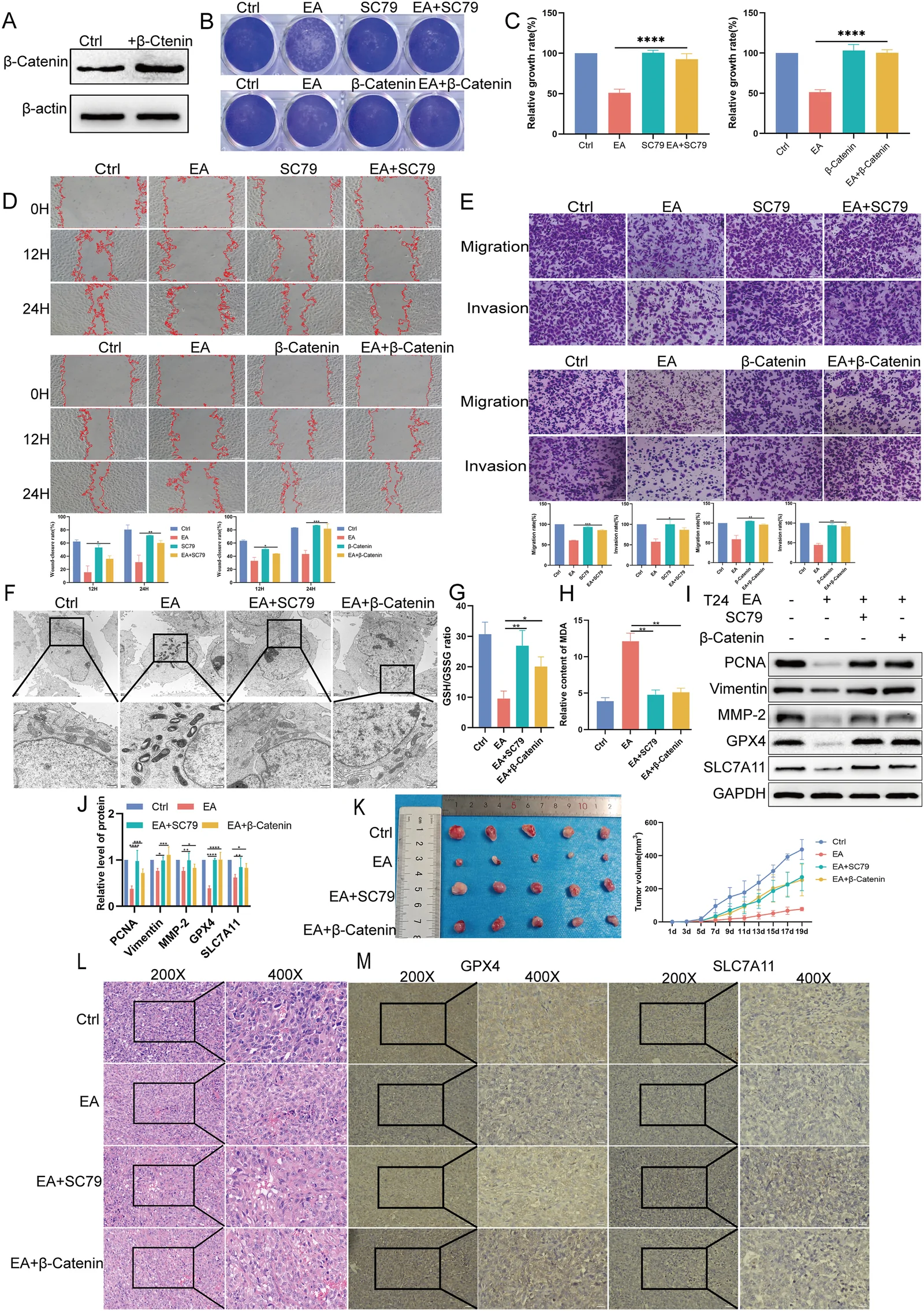
Echinocystic acid (EA) inhibits proliferation, invasion, and migration, while inducing ferroptosis in breast cancer cells via the AKT1/GSK3β/β-Catenin axis. **(A)** β-Catenin overexpression in T24 cells using adenovirus (Ad-β-Catenin) (*n* = 3). **(B, C)** Crystal violet staining (B) and CCK-8 assay (C) were used to assess the recovery of EA-induced cell viability inhibition by AKT1 agonist SC79 (10 μM) or Ad-β-Catenin (*n* = 3). **(D)** Wound healing assay was used to confirm the recovery of EA-induced migration inhibition by SC79 or Ad-β-Catenin. **(E)** Transwell assay was used to confirm the attenuation of EA's anti-migratory/invasive effects by SC79 or Ad-β-Catenin. **(F)** Transmission electron microscopy of mitochondrial ultrastructure of breast cancer cells after co-treatment of EA/SC79 or EA/Ad-β-Catenin (magnification: 8000 × and 25000 ×). **(G)** GSH and GSH/GSSG ratio determination of breast cancer cells after co-treatment of EA/SC79 or EA/Ad-β-Catenin (*n* = 3). **(H)** MDA content of breast cancer cells after co-treatment of EA/SC79 or EA/Ad-β-Catenin (*n* = 3). **(I, J)** Western blotting analysis (I) and quantification (J) of essential proteins in AKT1/GSK3β/β-Catenin pathway following co-treatment of EA/SC79 or EA/Ad-β-Catenin (*n* = 3). **(K)***In vivo* tumor image and growth curves (*n* = 5). **(L, M)** Representative images of hematoxylin–eosin staining (L) and immunohistochemical staining (M) of xenograft tumors (magnification: 200 × and 400 ×). The data are displayed as mean ± standard deviation. ∗*p* < 0.05, ∗∗*p* < 0.01, ∗∗∗*p <* 0.001, and ∗∗∗∗*p* < 0.0001.

### Synergistic effects of EA and GEM

Combination drug therapy is a clinically validated approach for improving treatment outcomes and reducing side effects through synergistic mechanisms. SynergyFinder analysis revealed a robust synergistic effect between GEM and EA, as reflected by a ZIP synergy score exceeding 10 (Fig. 8A and B). Based on Jin's formula, 20 μM of EA and 200 nM of GEM were identified as the optimal dosage combination, yielding a Q-value greater than 1.15 (Fig. 8C). Functional assays *in vitro* showed that the EA/GEM combination exerted significantly stronger anti-tumor effects than either agent alone, leading to enhanced suppression of cellular proliferation (Fig. 8D), migration, and invasion (Fig. 8E–G). Additionally, the EA/GEM combination also down-regulated proteins associated with proliferation and metastasis, including PCNA, Vimentin, and MMP2 (Fig. 8H). *In vivo*, EA/GEM combination constrained tumor volumes more substantially, when compared with mono-drug treatment (Fig. 8I), without significant body weight loss (Fig. 8J). Hematoxylin–eosin staining and immunohistochemical analyses provided further evidence of the superior therapeutic efficacy of the combination treatment, as evidenced by a more pronounced synergistic down-regulation of representative proteins associated with proliferation and migration/invasion (PCNA, Vimentin, and MMP2) (Fig. 8K and L). Thus, the results suggest a potential synergistic effect of the EA/GEM combination, which is more powerful in inhibiting BC cell growth.

**Figure 8:**
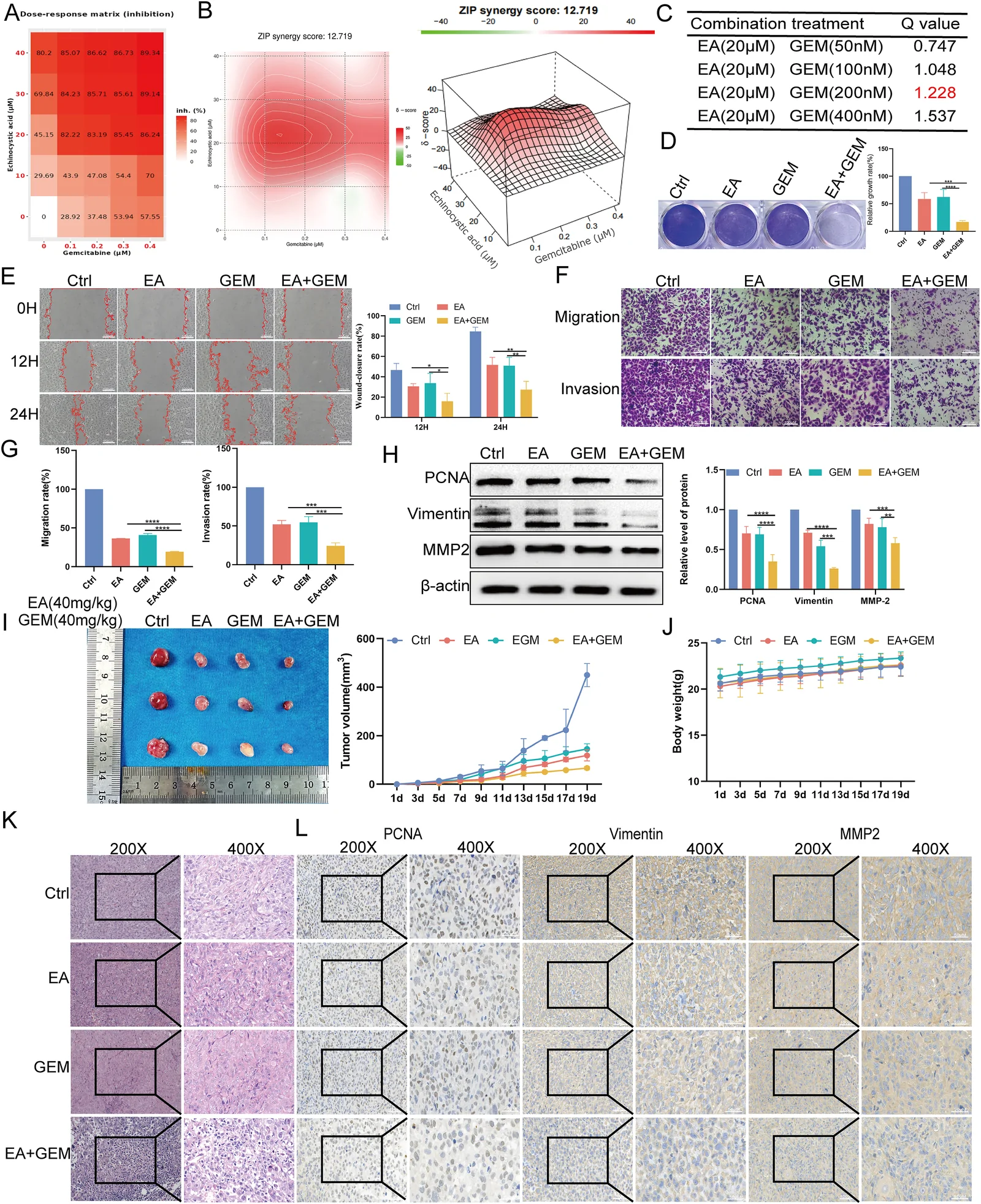
Synergistic anti-tumor effects of echinocystic acid (EA) and gemcitabine (GEM) in breast cancer cells. **(A, B)** Inhibition rate analysis (A) and ZIP synergy (B) of the EA/GEM combination were depicted by SynergyFinder computational prediction. **(C)** Jin's formula combination index quantification (*Q* > 1.125 at EA 20 μM + GEM 200 nM). **(D)** Crystal violet staining showed an enhanced growth inhibition of breast cancer cells after EA/GEM combination (*n* = 3). **(E)** Scratch assay demonstrated an enhanced inhibitory effect of EA/GEM combination on the migration of breast cancer cells (*n* = 3). **(F)** Transwell migration/invasion assay demonstrated an enhanced inhibitory effect of the EA/GEM combination on the migration and invasion of breast cancer cells. **(G)** Quantitative analysis of Transwell migration/invasion assay (*n* = 3). **(H)** Synergistic down-regulation of proliferation/metastasis markers by EA/GEM combination was determined by Western blotting (*n* = 3). **(I)***In vivo* tumor image and growth curves (*n* = 3). **(J)** Body weight monitoring during treatment (*n* = 3). **(K)** Representative images of hematoxylin–eosin staining of xenograft tumors (magnification: 200 × and 400 ×). **(L)** Representative images of immunohistochemical staining of xenograft tumors (magnification: 200 × and 400 ×). The data are displayed as mean ± standard deviation. ∗*p* < 0.05, ∗∗*p* < 0.01, ∗∗∗*p <* 0.001, and ∗∗∗∗*p* < 0.0001.

## Discussion

Natural products have long been recognized as an abundant resource for anti-cancer drug discovery due to their extensive chemical diversity, broad tumor-suppressing capability, and favorable safety and tolerability.^26^ Statistics record that over half of authorized anti-cancer drugs are natural products or their structurally optimized derivatives,^27^ directly underscoring their indispensable role in pharmaceutical development. Nevertheless, the pharmacological potential of natural products remains largely untapped.^28^ While several natural product-derived drugs such as paclitaxel and vincristine have shown efficacy in treating BC, there remains a pressing need for novel natural compounds that can effectively target this malignancy. Herein, we discovered that EA exhibits potent inhibitory activity against BC cells with a relatively excellent safety profile. At the mechanistic level, we elucidated that EA may target AKT1 and suppress its phosphorylation, thereby inhibiting AKT1/GSK3β/β-Catenin signaling and ultimately inducing ferroptosis.

Research into the anti-tumor pharmacological effects of EA is currently limited, with existing literature thus far confined solely to hepatocellular carcinoma cells (HepG2), promyelocytic leukemia cells (HL-60), non-small cell lung cancer cells (A549), and ovarian cancer cells (SKOV3 and A2780).^7^, ^8^, ^9^^,^^29^ However, it is notable that all studies consistently emphasized the significant anti-tumor potential of EA. Among all the aforementioned tumor cells, EA exhibited marked cytotoxic activity, effectively inhibiting cell proliferation. The results of the present investigation confirmed that EA also remarkably suppressed the viability of BC cells, thus reaffirming its tumoricidal efficacy. Furthermore, BC cells underwent extensive accumulation in the G1 phase upon EA treatment, alongside a reduction in Cyclin D1, which is a critical modulator governing the G1 to S phase transition.^30^ These results closely aligned with the prior work by Zhu et al, who clearly showed that EA caused G1 arrest and reduced Cyclin D1 expression in A549 non-small cell lung cancer cells. In fact, inducing G1-phase cycle arrest to inhibit tumor cell proliferation constitutes a precise mechanism by which lots of antineoplastic agents operate. Examples include commercially available pharmaceuticals such as trilaciclib and palbociclib,^31^^,^^32^ and natural products such as fucoidan and berberine.^33^^,^^34^ Therefore, EA may exert its anti-proliferative effect at least partly through triggering G1 arrest in BC cells. Apart from its uncontrollable growth, BC is distinguished by its significant metastatic capability, which is a substantial contributing factor to the poor prognosis.^35^ Consequently, inhibiting metastasis is of critical importance for improving patient survival. The acquisition of metastatic potential by tumor cells largely depends on epithelial–mesenchymal transition and the ability of MMPs to break down the extracellular matrix,^36^ which serve as core drivers of malignant progression in BC.^37^ To date, only one investigation has assessed the anti-metastatic properties of EA, demonstrating that it impaired the migration and invasion potential of non-small cell lung cancer cells.^8^ In line with these findings, we observed a reduction in the migratory and invasive potency of BC cells treated with EA, further highlighting its potential efficacy in inhibiting tumor cell metastasis. Furthermore, we discovered that EA significantly reversed epithelial–mesenchymal transition and down-regulated MMP2 protein level. This strongly indicates that EA holds potential therapeutic value in inhibiting the metastasis of BC cells. In summary, EA indeed exhibits excellent inhibitory activity against the proliferation, migration, and invasion of BC cells. While inhibiting BC cells effectively, EA also demonstrated preliminary safety. *In vitro*, EA exhibited no significant toxicity towards bladder epithelial cells and renal tubular cells, both of which originate from the urinary system. *In vivo*, EA did not appear to result in significant organ toxicity. Interestingly, we observed a marked increase in platelet count following EA administration, which was analogous to that of radix notoginseng saponins (another class of triterpenoids).^38^ Therefore, the platelet-elevating properties of EA may have potential applications in managing chemotherapy-induced thrombocytopenia. However, it should be noted that when used alone as an antineoplastic agent, EA may increase the risk of thrombosis.

The induction of apoptosis is one of the most well-established therapeutic strategies in tumor therapy, primarily achieved by activating apoptotic pathways or by inhibiting anti-apoptotic mechanisms in tumor cells.^39^ Numerous natural compounds, including ellagic acid, securinine, and echinatin, have been identified as effective apoptosis inducers in BC cells.^40^, ^41^, ^42^ Given prior studies that EA promoted apoptosis successfully in hepatocellular carcinoma cells, leukemia cells, and lung cancer cells,^7^, ^8^, ^9^ it was plausible to expect that it might also have a comparable impact on BC cells. However, contrary to this expectation, our research found that EA failed to induce apoptosis in BC cells, even at concentrations effective in suppressing proliferation. We speculated that this discrepancy may be attributable to at least one or more of the following reasons: i) Key pathogenic molecules and drivers are extremely different in tumor types. For instance, BC is frequently characterized by FGFR3 mutations,^43^ whereas hepatocellular carcinomas often exhibit TP53 and CTNNB1 alterations.^44^ Non-small cell lung cancer commonly harbors EGFR mutations,^45^ and leukemias are predominantly driven by chromosomal translocations (*e.g.*, BCR-ABL) and mutations in epigenetic modifiers.^46^ These differences may profoundly influence cellular responses to drugs. ii) Inducing apoptosis is merely one of the mechanisms by which anti-cancer drugs work. Actually, many natural products function primarily through non-apoptotic alternative pathways, such as antioxidant activity, autophagy induction, or triggering other forms of regulated cell death, rather than directly stimulating apoptosis.^47^, ^48^, ^49^ Therefore, we proposed that EA might initiate a non-apoptotic form of cell death in BC cells. This proposition was subsequently validated in the current study, with results indicating that EA can induce ferroptosis in BC cells.

It is noteworthy that our study found that EA induced ferroptosis in BC cells, as evidenced by ROS accumulation and mitochondrial shrinkage, features that were reversed by the ferroptosis inhibitor Fer-1. As a regulated cell death process that depends on iron and is driven by lipid peroxidation, ferroptosis has gained recognition as a novel anti-tumor mechanism.^50^ Currently, multiple clinically used agents, including artemisinin, statins, sorafenib, and sulfasalazine, have been confirmed to initiate ferroptosis.^51^ Notably, accumulating evidence indicates that natural products can also function as potent ferroptosis inducers. For instance, acevaltrate promoted ferroptosis in colorectal cancer cells by targeting PCBP1/2 and GPX4,^52^ whereas dendrobium triggered calcium/calmodulin-dependent ferroptosis in lung cancer cells.^53^ In BC cells, both evodiamine and baicalin have been shown to successfully induce ferroptosis.^54^ Therefore, natural product-induced ferroptosis offers a hopeful therapeutic modality against refractory malignancies. Our research discovered that EA effectively triggered ferroptosis in BC cells. This establishes the first confirmation of EA's potential to induce ferroptosis in tumor cells, thereby enriching our understanding of its anti-tumor action. Meanwhile, recent studies have shown that multiple ferroptosis-related proteins, including the ferroptosis inhibitor SLC7A11, are aberrantly expressed in BC tissues and that numerous ferroptosis-associated genes are closely correlated with the prognosis of patients with BC,^55^^,^^56^ highlighting the important biological role of ferroptosis in the initiation and progression of BC. In this context, our finding that EA induces ferroptosis in BC cells further supports the feasibility and translational potential of targeting ferroptosis pathways for the treatment of BC.

Our research indicated that EA interacted with AKT1 to inhibit its phosphorylation, thereby suppressing the AKT1/GSK3β/β-Catenin signaling pathway. As one of the AKT serine/threonine protein kinase family members, AKT1 serves as a central component within the canonical PI3K/AKT signaling pathway.^57^ AKT1 frequently exhibits hyperactivation and overexpression across various tumors.^58^, ^59^, ^60^ This dysregulation may not only facilitate tumor formation, but also suppress apoptosis and be associated with resistance to cancer treatment. Consequently, AKT1 has been established as a key target in anti-tumor therapy, and AKT1 inhibitors are a highly promising class of anti-cancer drugs with broad therapeutic potential across various malignancies.^61^^,^^62^ The AKT1/GSK3β/β-Catenin signaling pathway is a critical pathway activated by AKT1 that is intrinsically linked to tumor cell proliferation, migration, and invasion. Notably, this pathway has also been implicated in the modulation of ferroptosis.^63^, ^64^, ^65^ The critical molecular linchpin connecting the AKT1/GSK3β/β-Catenin pathway to the ferroptosis execution is the transcriptional regulation of SLC7A11 by β-Catenin.^25^ Specifically, β-Catenin interacts with TCF4 to form a transcription complex. This complex directly binds to the promoter sequences of SLC7A11 and GPX4, thereby promoting their expression and inhibiting ferroptosis.^66^ Our findings demonstrated that EA suppressed the AKT1/GSK3β/β-Catenin pathway to induce ferroptosis in BC cells, thus confirming the regulatory relationship between this pathway and ferroptosis. As a first-line treatment for BC, GEM frequently encounters issues with drug resistance, which may be associated with the protective activation of AKT1 in BC cells.^67^^,^^68^ Natural products targeting AKT1 can mitigate GEM chemoresistance, as seen with berberine^69^ and sulforaphane.^70^ Our combination experimental results indicated that EA and GEM could synergistically suppress the proliferation, migration, and invasion of BC cells, in line with previous studies on curcumin.^71^ Although the mechanism underlying this synergistic effect remains unclear, we hypothesized that it may be partially mediated through the inhibition of AKT1 by EA. Clinically, GEM remains a first-line or commonly used chemotherapeutic agent for BC and exerts its anti-tumor effects primarily by inhibiting DNA synthesis and inducing apoptosis. However, tumor cells may mount compensatory survival responses under GEM-induced stress, including activation of the PI3K/AKT pathway, which has been implicated in chemoresistance.^67^ Consistent with this notion, we observed that GEM increased AKT1 phosphorylation, whereas co-treatment with EA markedly attenuated this phosphorylation (Fig. S2), providing a plausible molecular basis for the enhanced anti-bladder-cancer activity of the combination.

## Conclusion

In summary, this study demonstrates that EA possesses potent anti-bladder cancer activity through suppressing the AKT1/GSK3β/β-Catenin pathway, thus inhibiting cell proliferation, invasion, and migration, while concurrently inducing ferroptosis. Furthermore, EA exhibits a favorable safety profile and shows synergistic efficacy with GEM, emphasizing its potential as a therapeutic agent for monotherapy or combination therapy in BC treatment. Naturally, this study has certain limitations. For example, it did not clarify in depth how EA induced ferroptosis by inhibiting the AKT1/GSKβ/β-Catenin signaling pathway, nor did it analyze the molecular mechanism underlying the synergistic effects of EA and GEM. In future work, several unresolved issues concerning EA's anti-cancer activity require further investigation, including i) identification of other potential molecules or signaling pathways that may also be associated with EA's anti-tumor potential; ii) analysis of its pharmacokinetics *in vivo*; iii) further assessment of its safety profile; and iv) confirmation of its anti-cancer activity in other tumors.

## CRediT authorship contribution statement

**Xueqian Han:** Writing – original draft, Project administration, Investigation. **Liping Xie:** Data curation, Methodology, Writing – review & editing. **Ziyun Li:** Visualization, Investigation. **Yajun Hou:** Visualization, Investigation. **Yunfeng He:** Supervision, Conceptualization. **Liqiong He:** Supervision, Funding acquisition, Conceptualization. **Jinyong Luo:** Supervision, Funding acquisition, Data curation, Conceptualization.

## Data availability

Data are available if requested.

## Funding

This research was supported by the Natural Science Foundation Project of Chongqing Science and Technology Commission (China) (No. CSTB2024NSCQ-MSX0340) and the funding of 10.13039/501100002865Chongqing Science and Technology Bureau (China) (No. CSTB2025KJCBPJ-KPZPYB5X0023).

## Conflict of interests

The authors declared no conflict of interests.
