# Supplementary material for: Echinocystic acid inhibits the growth of human bladder cancer cells by suppressing AKT1/GSKβ/β-Catenin signaling and synergizes with gemcitabine
Source: Genes Dis. 2026 Mar 19;14(2):102148. doi: 10.1016/j.gendis.2026.102148 (PMC13677775; doi:10.1016/j.gendis.2026.102148)
Supplement: Supplementary file 1 [file mmc3.pdf]

IACUC Approval Certification

Approval number:IACUC-CQMU-2024-0003

Project title:To explore the effect and mechanism of echinocystic acid on human bladder cancer cells

Funding source:National Natural Science Foundation of China (812740001)

Principal investigator:Jinyong Luo

Department:Laboratory Medical College of Chongqing Medical University

Application date:2023-10-23

Approval date:2024-01-02

All animal procedures to be employed in the project was approved by Institutional Animal Care and Use of Chongqing Medical University (IACUC-CQMU)。

Jun Yuan

Jun Yuan, MD
Chair, IACUC
Chongqing Medical University

重庆医科大学动物实验伦理审查同意书

批准编号:IACUC-CQMU-2024-0003

项目名称:探究刺囊酸对于人膀胱癌细胞的影响及机制

项目来源：国家自然科学基金（812740001）

项目负责人:罗进勇

负责人单位:检验医学院

申请日期:2023年10月23日

批准日期:2024年01月02日

本项目所涉及的动物实验方案经过重庆医科大学实验动物管理和使用委员会（IACUC-CQMU）审查，符合动物保护、动物福利和伦理原则，符合国家实验动物福利伦理的相关规定，同意按此方案进行实验。

重庆医科大学实验动物管理和使用委员会

2024年01月02日
