# Supplementary material for: Echinocystic acid inhibits the growth of human bladder cancer cells by suppressing AKT1/GSKβ/β-Catenin signaling and synergizes with gemcitabine
Source: Genes Dis. 2026 Mar 19;14(2):102148. doi: 10.1016/j.gendis.2026.102148 (PMC13677775; doi:10.1016/j.gendis.2026.102148)
Supplement: Supplementary file 2 [file mmc1.docx]

**Figure S1 EA does not induce apoptosis in BC cells. (A)** Hoechst staining of T24 and EJ cells. **(B)** Flow cytometry analysis of apoptosis in T24 cells.

**Figure S2 EA inhibits GEM-induced AKT phosphorylation in BC cells. (A)** Western blot analysis. **(B)** Immunohistochemical staining.
