# Supplementary material for: Echinocystic acid inhibits the growth of human bladder cancer cells by suppressing AKT1/GSKβ/β-Catenin signaling and synergizes with gemcitabine
Source: Genes Dis. 2026 Mar 19;14(2):102148. doi: 10.1016/j.gendis.2026.102148 (PMC13677775; doi:10.1016/j.gendis.2026.102148)
Supplement: Supplementary file 3 [file mmc2.docx]

**Table S1 Antibody Information**

| **Antibody name** | **Vendor (Catalog No.)** | **Species / Clone type** | **Dilution** |
| --- | --- | --- | --- |
| AKT1 Antibody | Bimake (A5023) | Rabbit monoclonal | WB 1:1000 |
| C-Myc Antibody | Bimake (A5011) | Rabbit monoclonal | WB 1:1000 |
| Cyclin D1 Antibody | Bimake (A5035) | Rabbit monoclonal | WB 1:1000 |
| E-cadherin Antibody | Affinity (AF0131) | Rabbit polyclonal | WB 1:1000 |
| GAPDH Antibody | Proteintech (60004-1-Ig) | Mouse monoclonal | WB 1:1000 |
| GPX4 Antibody | Proteintech (67763-1-Ig) | Mouse monoclonal | WB 1:1000  IHC 1:100 |
| GSK3β Antibody | Affinity (AF5016) | Rabbit polyclonal | WB 1:1000 |
| HRP-conjugated goat anti-mouse IgG | ZSGB-BIO (ZB-2305) | Goat polyclonal | WB 1:5000 |
| HRP-conjugated goat anti-rabbit IgG | ZSGB-BIO (ZB-2301) | Goat polyclonal | WB 1:5000 |
| MMP2 Antibody | Affinity (AF0577) | Rabbit polyclonal | WB 1:1000  IHC 1:100 |
| N-cadherin Antibody | Bimake (A5598) | Rabbit monoclonal | WB 1:1000 |
| PCNA Antibody | Selleck (F0018) | Rabbit monoclonal | WB 1:1000  IHC 1:1000 |
| Phospho-AKT1 (Ser473) Antibody | Affinity (AF8355) | Rabbit polyclonal | WB 1:1000  IHC 1:100 |
| Phospho-GSK3β (Ser9) Antibody | Affinity (AF2016) | Rabbit polyclonal | WB 1:1000  IHC 1:100 |
| Snail Antibody | Affinity (AF6032) | Rabbit polyclonal | WB 1:1000 |
| Vimentin Antibody | Selleck (AF7013) | Rabbit polyclonal | WB 1:1000  IHC 1:100 |
| xCT Antibody | Abmart (T57046) | Rabbit monoclonal | WB 1:1000  IHC 1:100 |
| β-actin Antibody | Huadingbio (10011) | Mouse monoclonal | WB 1:1000 |
| β-Catenin Antibody | Bimake (A5038) | Rabbit polyclonal | WB 1:1000  IHC 1:100 |
